# The Western Pacific Regional Framework to End TB: overview and critical reflection

**DOI:** 10.5588/ijtldopen.24.0608

**Published:** 2025-02-01

**Authors:** K.H. Oh, F. Morishita, K. Rahevar, R-P. Yadav, H.T.G. Tran, G.B Marks, M.C Raviglione, B.J. Marais

**Affiliations:** ^1^ World Health Organization Regional Office for the Western Pacific, Manila, Philippines;; ^2^ Burnet Institute, Melbourne, VIC, Australia;; ^3^ Centre for Multidisciplinary Research in Health Science (MACH), University of Milan, Milan, Italy;; ^4^ Sydney Infectious Diseases Institute (Sydney ID) and the WHO Collaborating Centre in Tuberculosis, University of Sydney, Sydney, NSW, Australia.

**Keywords:** tuberculosis, public health, health services accessibility, socio-economic factors, health equity

## Abstract

Despite notable progress, TB remains a critical public health challenge in the Western Pacific Region. To address this, the WHO developed the Western Pacific Regional Framework to End TB, which aligns with global health targets, such as the End TB Strategy and the Sustainable Development Goals. Here, we critically review the Framework, highlighting its strengths and ongoing challenges, with suggestions as to how it might adapt. Although the Framework offers a comprehensive strategy for reducing the TB burden, its success depends on effective implementation across diverse socio-economic contexts. Key obstacles include undiagnosed infectious TB cases, limited healthcare infrastructure, persistent inequities in access to TB services, and drug-resistant TB. The COVID-19 pandemic exacerbated these challenges, disrupting TB services and delaying progress towards 2030 targets. The Framework’s reliance on multisectoral partnerships and innovative technologies presents opportunities but requires substantial political commitment, sustained funding and system-wide health reforms. Additionally, gaps remain in addressing social determinants of TB. Ensuring equity, maintaining political will, and fostering international collaboration are essential to overcoming these barriers. Continuous evaluation and adaptation will be crucial in ensuring the Framework’s effectiveness in eliminating TB in the Region by 2030.

## INTRODUCTION

Despite substantial progress in recent decades, TB remains a significant public health concern in the Western Pacific Region (WPR). The international targets set by the World Health Assembly in 2014 and the latest revisions at the United Nations General Assembly High-Level Meeting on TB in 2023 are unlikely to be achieved by most countries. To guide and accelerate the TB response in the WPR, the WHO Regional Office for the Western Pacific developed a regional framework to end TB by 2030,^1^ with technical advice from the Regional Technical Advisory Group. It builds on a critical evaluation of the previous regional TB framework^2^ and incorporates feedback from member states and partners. The Framework was endorsed by the WHO Regional Committee for the Western Pacific in October 2021 and has since guided the regional TB control response. Here, we describe recent epidemiological trends, summarise the Framework’s key strategies, assess progress and challenges, and critically reflect on the ‘real life’ implementation of the regional Framework. The epidemiological and programmatic indicators were sourced from the WHO’s Global TB Report 2024^3^ and the global TB database.^4^

### Overview of TB epidemiology

In 2023, an estimated 10.8 million people developed TB worldwide, with nearly 20% of cases occurring in the WPR. In 2023, the region had an estimated 1.9 million people who developed TB (97/100,000 population) and 95,000 people with TB who died. Since 2015 and the launch of the End TB Strategy, the region has seen only a modest 3% reduction in the TB incidence rate and a 12% reduction in TB deaths by 2023 (Figure). Estimated TB incidence varies greatly among countries and areas of the region (Table 1). Five countries (China, Philippines, Viet Nam, Cambodia and Papua New Guinea) account for 94% of the total number of incident TB cases. The COVID-19 pandemic caused significant disruption to TB services, resulting in a 19% decline in case notifications in 2020 compared to 2019. However, TB case notifications returned to pre-pandemic levels in 2023, with 1.4 million cases notified. Despite this increase in notifications, estimated TB treatment coverage remained suboptimal at 74% in 2023, indicating that at least a quarter of all people with TB were missed by national TB control programmes. This includes people with TB who remain undiagnosed or unreported. National TB prevalence surveys have consistently demonstrated that a substantial proportion of people with bacteriologically confirmed pulmonary TB do not report symptoms suggestive of TB,^5,6^ and there is increasing evidence that people with asymptomatic TB contribute to disease transmission within communities.^7^ This has significant implications for current case-finding strategies and the global TB epidemic.^8^

**Figure.**
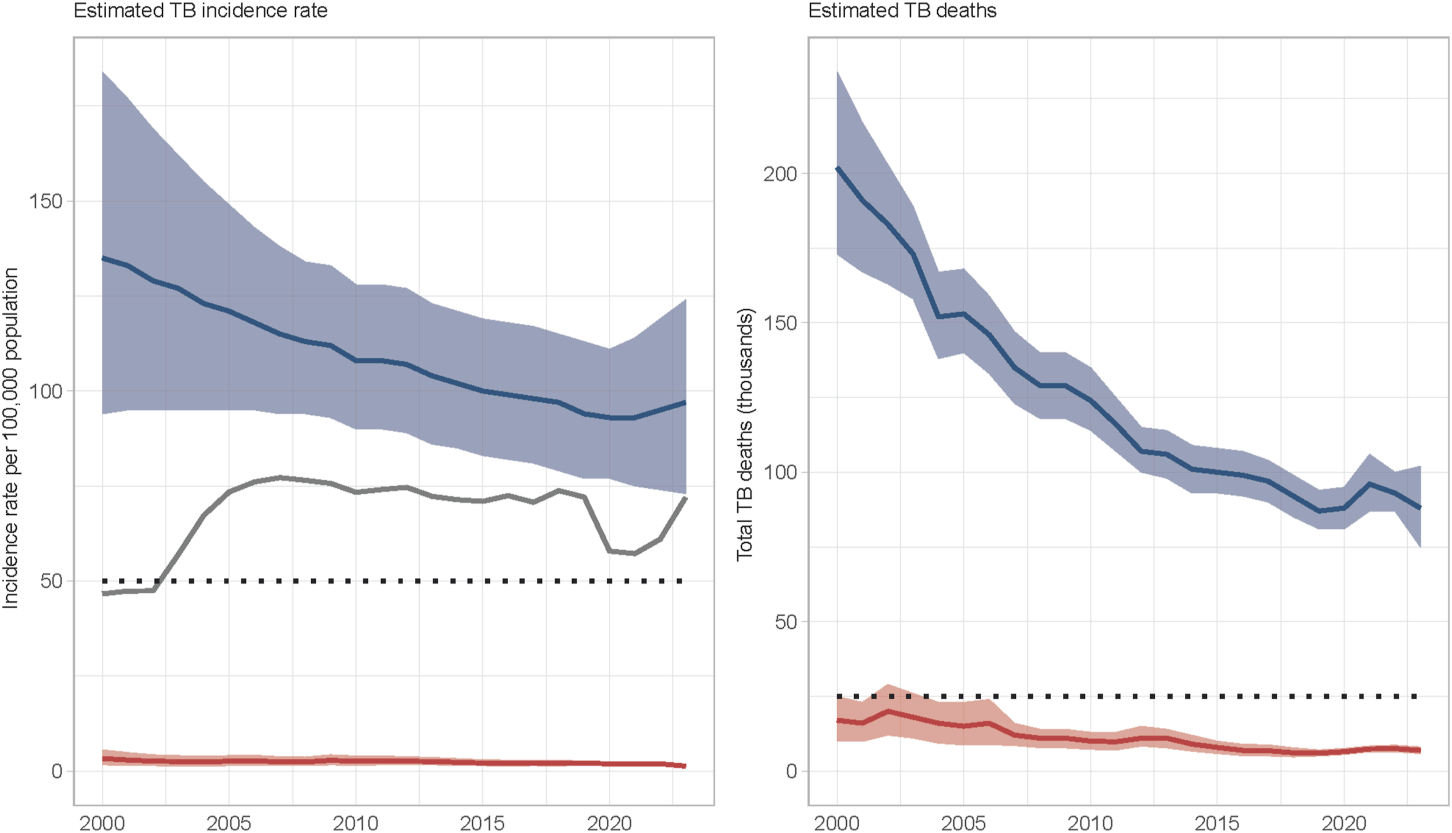
Estimated TB incidence rate and TB deaths, 2000–2023, Western Pacific Region. Estimated incidence and numbers of deaths are shown in dark blue and those among HIV-positive people in red. The horizontal dashed lines show the 2025 milestones of the End TB Strategy. Shaded areas represent uncertainty intervals. The grey solid lines show a case notification rate for new and relapse TB for comparison with estimated incidence rate.

**Table 1.** Estimated TB incidence and deaths in 2023, countries and areas of the Western Pacific Region.

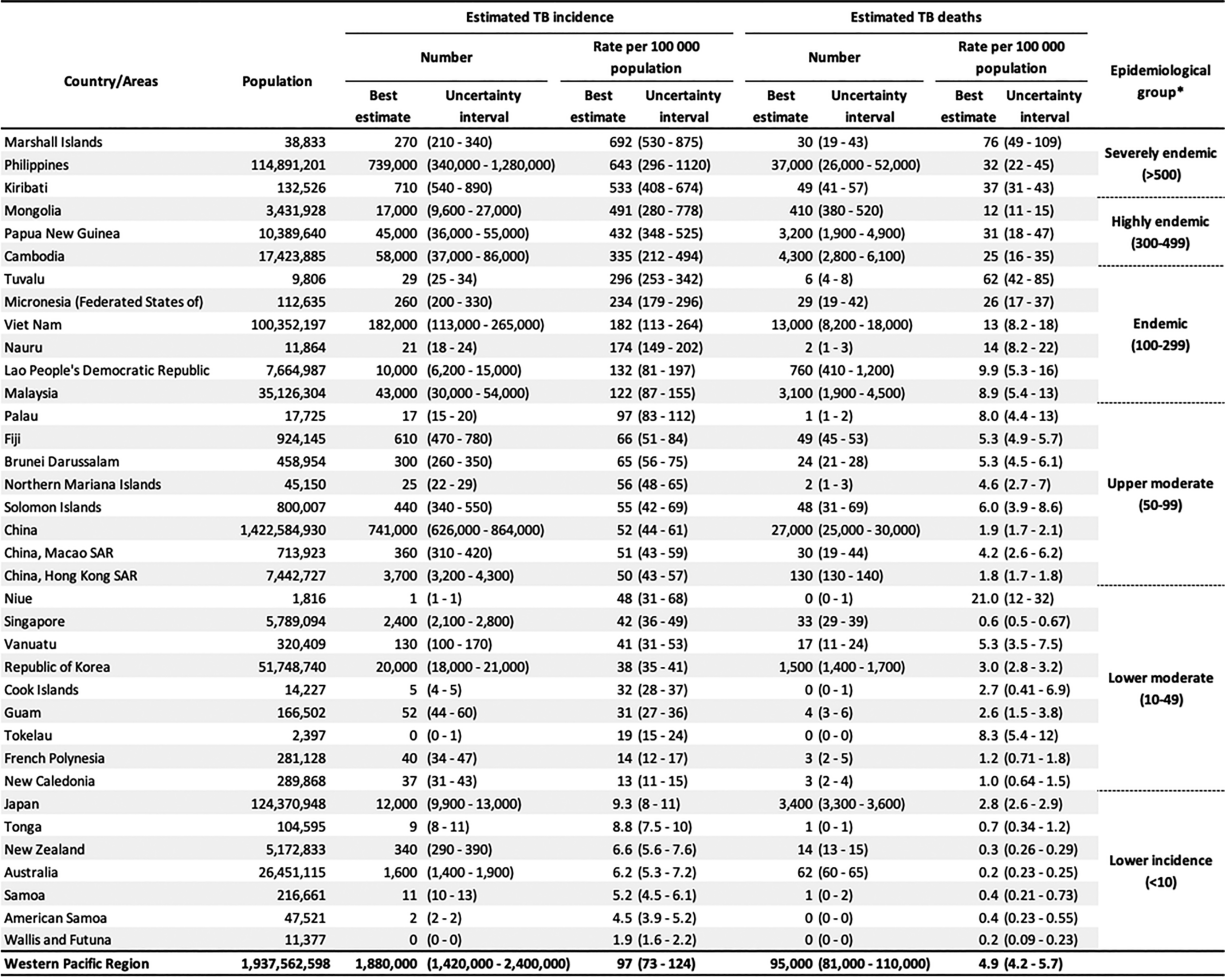

*Epidemiological categorisation is determined based on the estimated incidence rate per 100,000 population (best estimate).

In the region, the percentage of people newly diagnosed with pulmonary TB who were bacteriologically confirmed increased from 38% in 2015 to 58% in 2023, reflecting improved access and application of microbiological tests for TB diagnosis. The number of people diagnosed with MDR/RR-TB also increased by 60% (from 17,994 in 2015 to 29,425 in 2023). The percentage of those diagnosed with MDR/RR-TB who were enrolled on appropriate treatment increased from 76% in 2015 to 90% in 2023. The treatment success rate for new and recurrent TB cases remained high at nearly 90% between 2015 and 2019 but declined to 85% in 2022. Coverage of TB preventive treatment (TPT) among people living with HIV (PLHIV) has remained below 50%, although the number of people receiving TPT rose from 13,319 in 2016 to 21,412 in 2023. In 2023, estimated coverage of TPT among all household contacts was low at 5.5% (range: 5.3–5.6).

## FUNDAMENTAL TRANSITIONS IN THE WPR

Given its 10-year time horizon, the Framework specifically considered fundamental transitions and future challenges, such as major demographic, epidemiological and socio-economic shifts, in tailoring guidance to the diverse contexts within the Region.

### Demographic transition

TB among older adults poses significant challenges: in 2023, over 25% of TB cases were among those aged 65 and older, much higher than the global average of 13%. High proportions of adults aged 65 and older in total TB case notifications are reported in countries like Japan (67%), the Republic of Korea (58%), Hong Kong SAR China (52%). In countries that have experienced a substantial decline in the prevalence and incidence of TB (particularly the East Asian countries cited above), older cohorts who lived through the period of high TB prevalence have a higher prevalence of TB infection.^9^ Older people also have a higher propensity to progress from remote past TB infection to TB disease due to waning immunity with age, a high prevalence of comorbidities (e.g. cancer, diabetes, etc.) and the use of immunosuppressive treatments.^9^ Programmatic challenges include reaching older people for diagnosis and managing comorbidities.^9,10^ As the Region’s population ages, TB programmes must adapt to address these growing challenges effectively.

### Socio-economic development

Socio-economic development is linked to TB epidemiology: improved nutrition and reduced crowding have a beneficial impact on the transmission and incidence of TB,^11^ and improved health systems lead to better detection, diagnosis and treatment.^12,13^ Conversely, a high burden of TB has serious adverse socio-economic consequences for individuals, families and communities. Many countries in the region are experiencing rapid economic growth, which can improve health if the benefits are widely shared across society.^14^ If this occurs without people being left behind, it will potentially reduce the TB burden in the long term. Unfortunately, wealth inequality remains high and is increasing in many countries.^15^ While health service coverage increased, catastrophic expenditures have also risen dramatically,^16^ creating significant challenges for poor people suffering from TB. Health financing varies, with higher-income countries relying on public funding and middle-income countries increasingly dependent on domestic funding as external support declines. Rapid urbanisation (driven by rural poverty and increased economic opportunities in cities) complicates TB control, requiring urban TB programmes to adapt to diverse populations, multiple authorities and varied healthcare providers in rapidly expanding cities.

### Epidemiological transition

By 2030, non-communicable diseases (NCDs) are expected to be the leading cause of death in nearly all countries and areas of the WPR.^17^ However, COVID-19 has demonstrated that outbreaks of communicable diseases remain an ever-present threat, and TB remains the leading infectious disease killer on the planet, with many comorbidities enhancing TB vulnerability and TB disease increasing the NCD burden.^18,19^ The rising prevalence of NCDs, such as diabetes mellitus (which compromises the immune response), along with risk factors (such as tobacco use, harmful alcohol consumption, poor diets and physical inactivity), will heighten the risk of TB infection and the progression to active TB. This trend could lead to reduced resource allocation and lower prioritisation of communicable diseases, including TB, posing significant challenges for TB control.

## CHALLENGES TO REGIONAL TB CONTROL AND ELIMINATION

Building on extensive situational analysis, critical assessment of preceding frameworks^2^ and considering future challenges, the Framework focused on four fundamental layers to guide an effective regional response (see Supplementary Data Figure S1).

### First layer: TB care and control challenges (TB-specific)

TB care faces significant challenges in three key areas: case finding, quality of care and prevention. In 2023, an estimated 26% of TB cases and 60% of MDR/RR-TB cases were undiagnosed or unreported. In part, this is due to inequitable access to services and weak health systems, but it is also due to the absence of symptoms in many people with TB. Delays in diagnosis and treatment compromise quality of care, inadequate use of WHO-recommended diagnostics, and poor treatment outcomes, especially for DR-TB.^20^ Prevention efforts are hindered by the lack of a fully effective vaccine, gaps in infection control, slow progress in providing TPT, and the risk of re-infection progressing to TB disease in people who have completed TPT in settings with uncontrolled TB transmission. In high-incidence settings, the presence of many people with infectious TB sustains ongoing transmission and limits the opportunities for effective prevention.

### Second layer: health system challenges (beyond TB, but within the health sector)

Challenges within health systems are categorised into four areas: lack or incomplete universal health coverage (UHC), insufficient collaboration with other programmes and systems to address TB risk factors and comorbidities, limited research and innovation capacity and investments, and insufficient community and civil society engagement. Many countries struggle with access to high-quality primary healthcare due to insufficient infrastructure, financing and human resources, leading to impediments to TB diagnosis and treatment. Collaboration remains underdeveloped and fragmented among different health programmes to address TB-related health risks, including undernourishment, diabetes, smoking, alcohol use and HIV infection. Although TB research has grown, significant gaps remain in operational research and local innovations.^21^ Additionally, community engagement is limited by a lack of platforms, especially for national programmes, to involve affected communities and resources to support community-led TB care and prevention efforts.

### Third layer: challenges related to the social determinants of TB (‘Health beyond health’)

Social determinants, such as poverty, socio-economic disparities, poor nutrition and inadequate living conditions, significantly influence TB risk and disease progression. In the national TB patient cost surveys conducted in high TB-burden countries of the WPR, 34–92% of TB-affected families reported catastrophic costs.^4,22^ Despite free TB services, patients often face catastrophic costs driven by non-medical expenses and income loss incurred prior to and during TB treatment, especially among poor households and those with drug-resistant TB or HIV co-infection.^22^ Insufficient or lack of social protection mechanisms exacerbate these issues. TB is closely linked to at least 12 Sustainable Development Goals (SDGs) that represent essential human development sectors, highlighting its broad socio-economic impact. The disease is more prevalent among those with low socio-economic status, who experience higher exposure to TB risk factors such as under- and mal-nutrition and poor living conditions. Unregulated urbanisation, climate change, the consequent massive displacement of populations, and political instability are expected to exacerbate these issues, increasing the challenges associated with migration and urban health. Addressing these challenges requires a holistic approach with strong multisectoral involvement and accountability.

### Fourth layer: governance and accountability challenges (overarching)

The overarching governance and accountability challenges for TB control include inadequate financing, weak coordination mechanisms, insufficient accountability and emergency planning. The End TB Strategy requires significant financial resources for implementation and research, yet historical underfunding hampers progress. Since 2020, funding available for TB prevention, diagnosis and treatment services in the WPR has not increased substantially.^4^ Effective TB control also demands coordination across multiple sectors, but competing priorities and a lack of multisectoral accountability often hinder current efforts. Additional accountability gaps exist as global commitments are not always translated into actionable policies at the national level. Finally, many TB programmes lack contingency plans for emergencies and natural disasters, leaving them vulnerable.

## REGIONAL FRAMEWORK

The regional Framework has the same objectives as the global End TB Strategy and is closely aligned with and tied to the SDGs (Supplementary Data Figure S2). Aiming for a TB-free WPR, the Framework incorporates a human rights-based approach and strives to reduce the TB incidence rate by 80%, cut TB deaths by 90% by 2030 compared to 2015, and eliminate catastrophic costs. It is aligned with the WPR’s initiative, which integrates TB care and prevention efforts into four key regional priorities: 1) health security, including antimicrobial resistance; 2) NCDs and ageing; 3) climate change, the environment and health; and 4) reaching the unreached. The Framework connects with targeted strategies to address these priorities, emphasising efforts to reach high-risk groups and the most vulnerable and underserved communities. The Framework’s approach is characterised by a country-specific, multisectoral partnership model and operates through several key modalities: 1) adopting a systems approach to improve service delivery; 2) promoting information-driven actions; 3) implementing strategic communication and change management; and 4) fostering innovation and rapid adoption of new methods. The Framework outlines key action areas: 1) strengthening essential TB functions (within TB); 2) building health system foundations (within health); 3) promoting health beyond health (beyond health); and 4) governance and accountability (overarching) (Box). The overarching aim is to guide national strategic planning for TB. It is intended to serve as a foundational tool for developing national plans and fostering collaboration across sectors, both within and beyond the health field, to implement the End TB Strategy in a way that is tailored to each country’s context. WHO and all relevant stakeholders are expected to support countries on their path to achieving the goal of eliminating TB in the WPR by 2030.

Box. Proposed actions of the WPR Framework to End TB.

1 To tackle TB-specific challenges, it calls for bolstering essential TB functions by:
a) ensuring timely diagnosis and reporting of TB across all population groups, especially the most vulnerable;
b) delivering equitable, people-centred TB care through decentralised care models, empowering affected individuals and families, integrating treatment adherence measures, and providing comprehensive palliative and end-of-life care; and
c) preventing TB transmission by enhancing infection control, expanding TB prevention treatment, and promoting bacille Calmette-Guérin (BCG) vaccination in high-prevalence areas
2 To address broader health system challenges, the Framework advocates for:
a) a systems approach towards achieving UHC by enhancing coverage attributes;
b) improving collaboration with other health programs, as well as with clinicians and professional organisations, to manage TB risk factors and comorbidities;
c) developing or strengthening national research networks; and
d) ensuring active involvement of community and civil society organisations
3 To address issues beyond the health sector, the Framework emphasises the importance of:
a) enhancing social protection mechanisms; and
b) encouraging a whole-of-government and whole-of-society approach.
4 To improve governance and accountability, the Framework recommends:
a) ensuring sustainable and adequate financing;
b) developing TB-sensitive policies and effectively translating these policies into practice;
c) strengthening management and coordination; and
d) effectively managing TB in emergency situations

WPR = Western Pacific Region; UHC = universal health coverage.

## CRITICAL REFLECTION

Implementing a regional framework to end TB involves navigating various challenges inherent to TB control and elimination. These challenges are amplified by socio-economic disparities, varying healthcare infrastructures and the unique epidemiological profiles across countries. The COVID-19 pandemic has highlighted the vulnerability of TB services to crises, reinforcing the need for resilient, scalable approaches. To meet the 2030 targets set by the End TB Strategy, it is essential to address TB control and elimination through four critical layers: strengthening essential TB functions, building robust health system foundations, addressing social determinants of health (‘health beyond health’), and enhancing governance and accountability. Each layer requires a tailored approach that focuses on TB-specific interventions and incorporates broader health system strengthening, social protection and committed political leadership. The following critical reflection delves into these layers, exploring both the challenges and potential solutions to realise the ambitious goals of the Regional Framework, offering a roadmap to effectively eliminate TB across the diverse contexts within the Region (Table 2).

**Table 2.** Critical reflection of the WPR Framework to End TB – implementation challenges and possible solutions.

Key point	Implementation challenges	Possible solutions
First layer: strengthening essential TB functions		
Expanding active case-finding	Limited impact of traditional case-finding strategies on reducing transmission and incidence Most TB transmission occurs outside traditional risk groups, often by asymptomatic individuals Resource-intensive population-based active case finding needed using radiology or NAAT	Integrate TB screening with broader health services to enhance sustainability Employ digital health tools, telemedicine, and portable diagnostics for remote area case finding
Sustaining TB services amidst crises	COVID-19 disrupted TB services, reducing diagnoses and treatment. Maintaining TB services during crises remains challenging without integrated, resilient systems	Adopt models like the Philippines’ integration of TB and COVID-19 services Utilise digital health and robust supply chain management Maintain investment and vigilance, and keep TB on the public health agenda
Second layer: building health system foundations		
Emphasis on system strengthening	System-wide reforms may divert resources from TB, potentially reducing access to TB diagnostics and treatment under UHC, as seen in Viet Nam	Balance system strengthening with TB-focused interventions to ensure accessible and free TB care under UHC
Potential for innovation and research	Innovation in diagnostics, treatments, and vaccines is vital but lacks clear strategies in low-resource countries Limited access to new technologies due to affordability issues	Foster public-private partnerships to support affordable innovation Develop sustainable funding mechanisms and encourage collaboration for research in low-resource settings
Third layer: promoting health beyond health		
Comprehensive multisectoral strategy	Frameworks focused on single diseases may lack sustainability and fail to address broader health needs effectively Cross-sector coordination is challenging without strong political support	Develop integrated accountability frameworks that support multiple diseases and primary healthcare as a whole Strengthen political leadership and intersectoral frameworks
Equity, access, and social protection	Equity in TB care is difficult in high-burden countries with limited infrastructure Lack of financial protection leaves TB-affected households vulnerable to economic hardship	Strengthen financial protection mechanisms for TB patients, including incentives and compensations Improve access to care in underserved regions to ensure equitable treatment coverage
Overarching layer: governance and accountability		
Critical elements for effective local implementation	Effective implementation needs local political support and tailored situational analysis. Identifying 'best value' interventions for each local context is complex	Promote local political commitment through advocacy and engagement Prioritise context-specific interventions to maximise resource efficiency and impact
Funding for TB control and elimination	Persistent funding gaps as external support declines, especially in high-burden countries Donor dependency weakens local programme ownership and sustainable progress	Increase domestic prioritisation of TB within national health budgets Transition to long-term, sustainable funding solutions by building local financial independence and reducing reliance on external donors

WPR = Western Pacific Region; NAAT = nucleic acid amplification test; UHC = Universal Health Coverage.

### Strengthening essential TB functions

#### Expanding active case finding through integrated approaches

Traditional case-finding strategies (including passive case finding and targeted active case finding) have limited epidemiological impact in high TB burden settings, as a certain proportion of transmitters are asymptomatic or outside typical risk groups. This underscores the need for population-based active case finding using symptom-agnostic tools like radiology or molecular tests, which are proven to reduce community transmission.^23^ Although essential, population-wide active case finding is costly and challenging to scale, a long-term economic analysis may reveal that high initial costs yield substantial savings, underscoring the need to explore efficient deployment methods across varied settings. Data on cost-effectiveness and scalability are critical to optimising active case finding in resource-limited, high TB incidence areas. A practical approach is integrating TB screening into broader health services, using innovative tools (such as portable AI-enabled X-rays and molecular testing) to empower peripheral health workers. Countries, including Mongolia and the Pacific island countries, have shown the potential of such integrated models.^24^ Combining TB screening with other services and using telemedicine for real-time support offers a scalable, cost-effective solution. National TB programmes should advocate for these integrated models, securing political and international backing for sustainable TB control strategies.

#### Sustaining TB services amidst crises

The COVID-19 pandemic significantly disrupted TB services, impacting diagnosis, treatment and prevention efforts.^25,26^ This led to a decline in TB case notifications and a rise in TB-related mortality and likely contributed to increased community transmission and drug resistance. In many countries, TB notifications have recovered to pre-pandemic levels. The response to COVID-19 in the Philippines provides key lessons in maintaining TB services during crises.^27^ By adapting its health systems, integrating TB and COVID-19 services, and ensuring uninterrupted supply chains, it was possible to prioritise TB.^27^ Digital health solutions and rapid policy changes also helped mitigate the pandemic’s impact. The challenge is to sustain this momentum and ensure TB remains a public health priority during future emergencies. Continued vigilance and investment in TB care are vital to achieving the 2030 targets, and governments must build on progress to prevent setbacks and remain on track for TB elimination.

### Building health system foundations

#### Emphasis on system strengthening

The Framework’s focus on strengthening health systems as a foundation for TB care aligns with the global push towards UHC, aiming to build sustainable infrastructure for TB and other health challenges. A strong health system is vital to ensuring access to quality TB care, from prevention to treatment. However, paradoxically, in high TB burden countries of the Region (such as China, Mongolia, the Philippines and Viet Nam), the introduction of social health insurance under UHC has, in some instances, reduced access to free TB diagnostics and treatment.^28,29^ Coverage gaps and delays have emerged, posing important challenges when integrating TB care into broader health reforms is not sensitive to specific TB response requirements. Although system-wide reforms are essential for long-term sustainability, they must not divert resources from urgent TB-specific needs. Balancing health system strengthening with a focused effort on TB diagnosis and treatment is crucial to ensure free and reliable access to essential services without compromising TB goals.

#### Potential for innovation and research

Innovation in tools, diagnostics, and treatments is clearly essential to meeting the ambitious targets of the End TB Strategy. Success depends on developing and adopting new technologies (such as vaccines, point-of-care diagnostics and shorter treatment regimens), particularly in high-burden settings.^30^ To advance these goals, concrete strategies are needed to foster regional research and development, especially in countries with limited capacity. Multinational partnerships that span the entire spectrum of low to high TB incidence settings are crucial to developing scalable and adequately tailored solutions. Collaboration between high-incidence and low-incidence countries within the WPR can provide opportunities for shared learning and innovation.^31^ Also, ‘South-South’ partnerships, where countries with similar challenges collaborate, are particularly beneficial for addressing common barriers. Securing sustainable funding remains a challenge, and the Framework should be an inspiration for countries to better outline strategies for attracting regional investment, including private sector incentives and dedicated funding mechanisms. Public-private partnerships are key, but the Framework needs more guidance on ensuring that new technologies are accessible and affordable in low-resource settings.

### Promoting health beyond health

#### Comprehensive multisectoral strategy

The Framework rightly highlights that TB is not just a medical issue but also a social and economic one, thriving in conditions like poverty, overcrowded housing, gender inequities, inadequate nutrition and limited education. Addressing these social determinants is vital for TB elimination, but it should not be seen as a prerequisite for TB control since progress through well-conducted and targeted health interventions is equally essential and cannot wait for societal change. Effective TB programmes are essential to ensure proper care, reduce suffering and prevent deaths. Countries such as China, the Philippines, and Viet Nam have developed multisectoral accountability frameworks for TB.^32^ However, having a separate multisectoral framework dedicated to one disease may not be feasible or sustainable in the long run. A more practical approach would be to develop multisectoral accountability frameworks that support all major diseases and primary healthcare (PHC). This integrated approach aligns with the core elements of PHC– multisectoral actions, community empowerment, and integrated primary care services – ensuring that health interventions are robust and sustainable across various health challenges. Strong political leadership is key to prioritising TB and fostering collaboration across sectors for PHC. Although cross-sectoral commitment is valuable, TB programmes must push forward with health-specific interventions. Proactive leadership and well-structured intersectoral frameworks can support TB control by overcoming socio-economic barriers to care.

#### Equity, access and social protection

The Framework emphasises equitable access to TB services, particularly for vulnerable and underserved populations, which is critical in the WPR, where TB burdens and health system capacities vary. Vulnerable groups (such as those in poverty, remote areas or marginalised communities) are often the most affected by TB. Achieving equity requires medical interventions and removing barriers to care, including access to adequate nutrition and food supplements.^33^ In high TB burden countries with weaker health systems, limited infrastructure can hinder the Framework’s comprehensive strategies, potentially widening the gap between well-equipped and less-equipped nations. The Framework could offer clearer guidance on tailoring interventions and fostering international collaboration and funding to help resource-limited countries close this gap. Although the Framework recognises the importance of social protection in TB elimination, implementation remains a challenge, particularly in high-burden countries. TB-affected households often face financial hardship due to income loss during treatment, and these costs are not always covered by UHC. Strengthening financial protection and involving people with lived TB experience in policy design is key to addressing these issues. Compensation for those undergoing treatment, integrated into existing social protection systems, would help ease economic burdens and support TB elimination efforts, especially in resource-limited settings.

### Governance and accountability

#### Critical elements for effective local implementation

For effective implementation of the regional TB framework, local priorities must be based on a detailed situational analysis. This involves understanding each region’s unique challenges (such as TB prevalence, demographics and social determinants of health). Thorough analysis enables local authorities to identify pressing issues and allocate resources where they will have the greatest impact. Assessing ‘best value’ interventions is also crucial, as not all are equally effective in every context and must be tailored to local needs. For example, regions with high rates of DR-TB may require advanced diagnostics and optimised regimens that enhance case detection and treatment success, whereas areas with low health literacy might benefit more from educational programmes and collaborative efforts with civil society and advocacy groups to address stigma and promote adherence. Prioritising interventions that offer the best value ensures efficient and effective use of resources. Strong local political buy-in is essential for success. Political leaders play a vital role in prioritising health issues, securing funding, and mobilising community support. Their commitment ensures that TB care remains a priority and that interventions are sustained. Political support also helps overcome implementation barriers, fostering ownership and accountability crucial for long-term success.

#### Funding for TB control and elimination

A key issue highlighted in the Framework is the ongoing funding gap for TB control and elimination, particularly in high TB incidence countries. While the Framework emphasises the need for adequate financial resources to support TB services, insufficient funding remains a major obstacle to achieving its ambitious targets. Many TB-endemic countries, especially those transitioning to middle-income status, are experiencing a reduction in external funding sources (such as the Global Fund), with increasing reliance on domestic financing.^34^ However, domestic funding for TB often competes with other urgent health priorities, which limits the resources available for TB programmes. Additionally, donor-driven targets focus on short-term outcomes rather than long-term, sustainable solutions for epidemic control. This approach can undermine efforts to build a strong, self-reliant national TB programme, as dependency on donor funding weakens local ownership and commitment to long-term strategies. Strengthening domestic funding mechanisms, alongside continued international support, is crucial for the financial sustainability of TB services. To address this challenge, countries must prioritise TB within their health budgets and explore innovative funding mechanisms to guarantee uninterrupted and comprehensive TB care. Without adequate and sustained investment, the Framework’s goal of TB elimination by 2030 remains at serious risk.

## CONCLUSION

The WPR Framework to End TB 2021–2030 presents a comprehensive strategy aimed at eliminating TB through a multisectoral approach aligned with global health goals. It could serve as a useful template for other WHO regions. However, its success relies on effective implementation, addressing resource limitations, ensuring equitable access and securing strong political commitment. Challenges like future emergencies, exemplified by the COVID-19 pandemic, underscore the need for resilient health systems. Innovation in diagnostics and treatments, supported by international collaboration and sustainable funding, is crucial for achieving the 2030 targets. Front-loaded investment, international and domestic collaborations, and strong regional leadership are essential for maintaining momentum towards TB elimination, along with the need for innovative strategies and more effective tools to significantly reduce community-level transmission, which perpetuates the epidemic.

## Supplementary Material


