# Supplementary material for: The Western Pacific Regional Framework to End TB: overview and critical reflection
Source: IJTLD Open. 2025 Feb 1;2(2):64–72. doi: 10.5588/ijtldopen.24.0608 (PMC11827672; doi:10.5588/ijtldopen.24.0608)

http://dx.doi.org/10.5588/ijtldopen.24.0608

The Western Pacific Regional Framework to End TB: overview and critical reflection

Supplementary Figure S1. Unpacking the different 'layers' challenging Regional TB control and elimination efforts

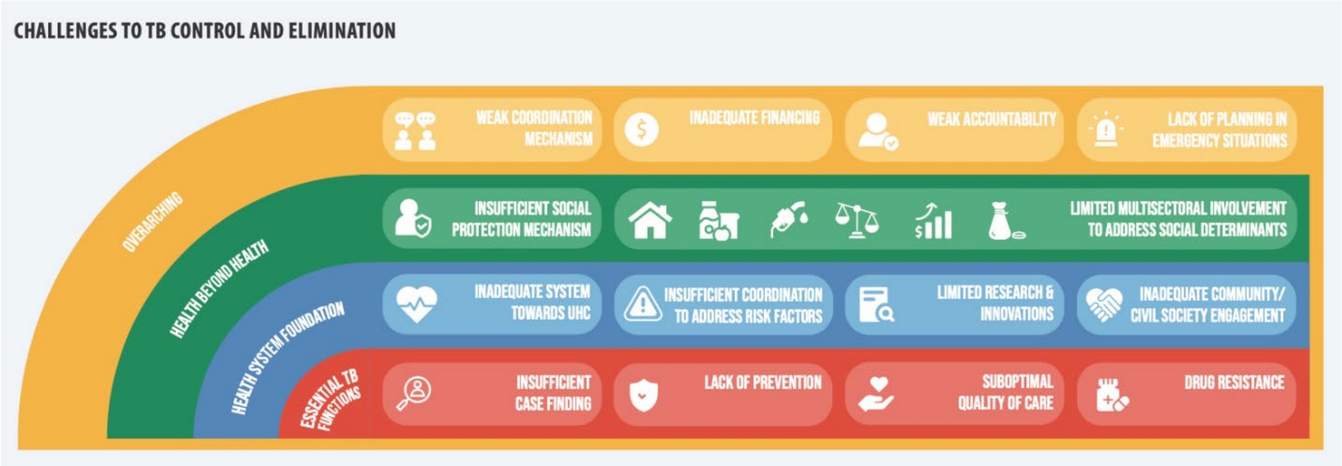

Supplementary Figure S2. Principles, approaches, action domains and operational modalities identified in the Western Pacific Regional Framework to End TB

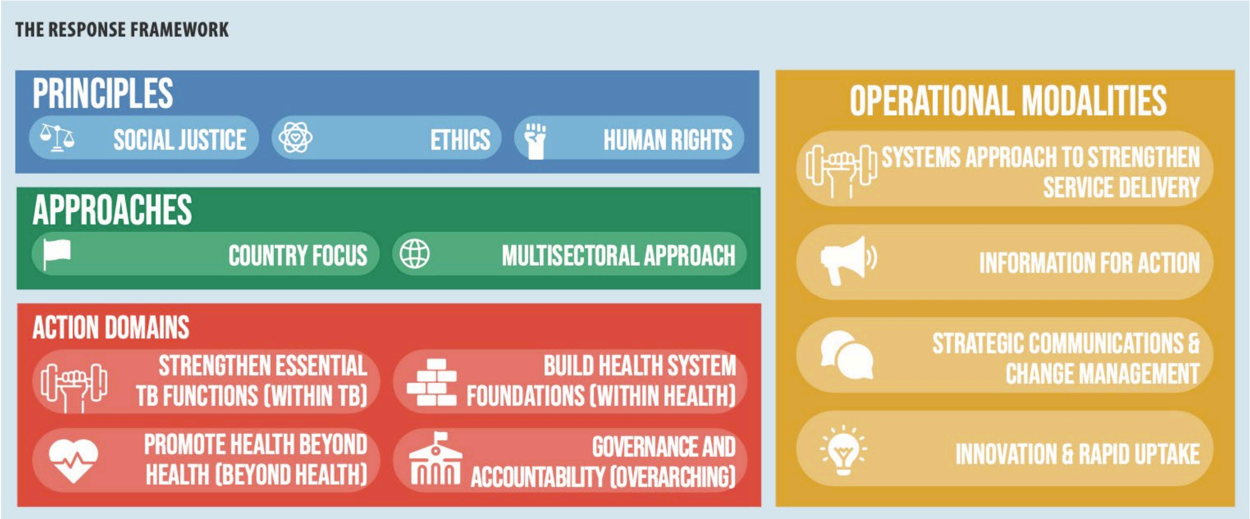

Supplement: Supplementary file 1 [file ijtldopen24_0608_supplementarydata1.pdf]
